# Chronic subthalamic nucleus deep brain stimulation reduces pathological TrkB aggregates in a Parkinson’s disease rat model

**DOI:** 10.1186/s40035-025-00472-x

**Published:** 2025-02-20

**Authors:** Tobias Petschner, Katarina Hofman, Jia Zhi Chen, Thomas Andreska, Daniel Wolf, Susanne Knorr, Robert Blum, Muthuraman Muthuraman, Uwe Gbureck, Jens Volkmann, Michael Sendtner, Chi Wang Ip

**Affiliations:** 1 Department of Neurology, University Hospital of Würzburg, Josef-Schneider-Straße 11, 97080 Würzburg, Germany; 2 Institute of Clinical Neurobiology, University Hospital of Würzburg, Versbacherstraße 5, 97078 Würzburg, Germany; 3 Department for Functional Materials in Medicine and Dentistry, University Hospital of Würzburg, Pleicherwall 2, 97070 Würzburg, Germany

Parkinson’s disease (PD), the second most common neurodegenerative neurological disease, is characterized by dopaminergic neuron loss in the substantia nigra (SN) and Lewy body formation. Subthalamic nucleus deep brain stimulation (STN-DBS) effectively treats motor symptoms like tremor and dyskinesia in PD. In addition to its acute effect on the motor symptom networks, there is an ongoing debate on a potential disease-modifying neuroplastic or even neuroprotective effect of chronic STN-DBS, and the underlying mechanisms remain elusive. Plasticity-associated factors, such as brain-derived neurotrophic factor (BDNF) and its receptor tropomyosin receptor kinase B (TrkB), can play important roles in retuning neural networks. Dopamine deficiency in PD striatum causes reduced sensitivity of dopamine receptor D1-expressing medium spiny neurons (dMSNs) to BDNF, leading to intracellular TrkB clustering. This highlights a mechanism relevant to PD pathogenesis and treatment [1].

In this study, we examined the formation of pathological TrkB clusters in MSNs of progressive AAV A53T human α-synuclein (hαSYN) PD rats and assessed the effect of chronic STN-DBS on these clusters (see Supplementary Material and Methods for experimental details). By co-staining DARPP-32 (dopamine and cyclic AMP regulated phosphoprotein of 32 kDa) with TrkB, we identified TrkB clusters in striatal MSNs, mainly in the lesioned hemisphere of unilateral hαSYN rats (Fig. 1a–c). By comparing hαSYN rats with increasing degrees of pathological severity induced by escalating concentrations of viral vector (low-c, middle-c and high-c), we observed TrkB cluster formation exclusively in the middle-c and high-c groups (Fig. 1c). The absence of TrkB clusters in the low-c hαSYN animals suggests that a certain threshold of disease severity and subsequent deficits in dopaminergic projections is required for the induction of TrkB accumulation. Despite similar cluster densities between the middle-c and high-c hαSYN groups, the high-c hαSYN rats exhibited significantly larger clusters (Fig. 1d). Notably, the TrkB cluster quantity exhibited an inverse correlation with dopaminergic tyrosine hydroxylase^+^ (TH^+^) terminals in the dorsolateral striatum (Fig. 1e, upper panel). The upper 95% confidence interval (CI) of the frequency distribution at 0.392 indicated a striatal TH^+^ terminal deficit of 60% as threshold for the development of TrkB clusters (Fig. 1e, lower panel). These results demonstrate that the development of TrkB clusters in the striatum of PD rats correlates with a loss of dopaminergic innervation.

**Fig. 1:**
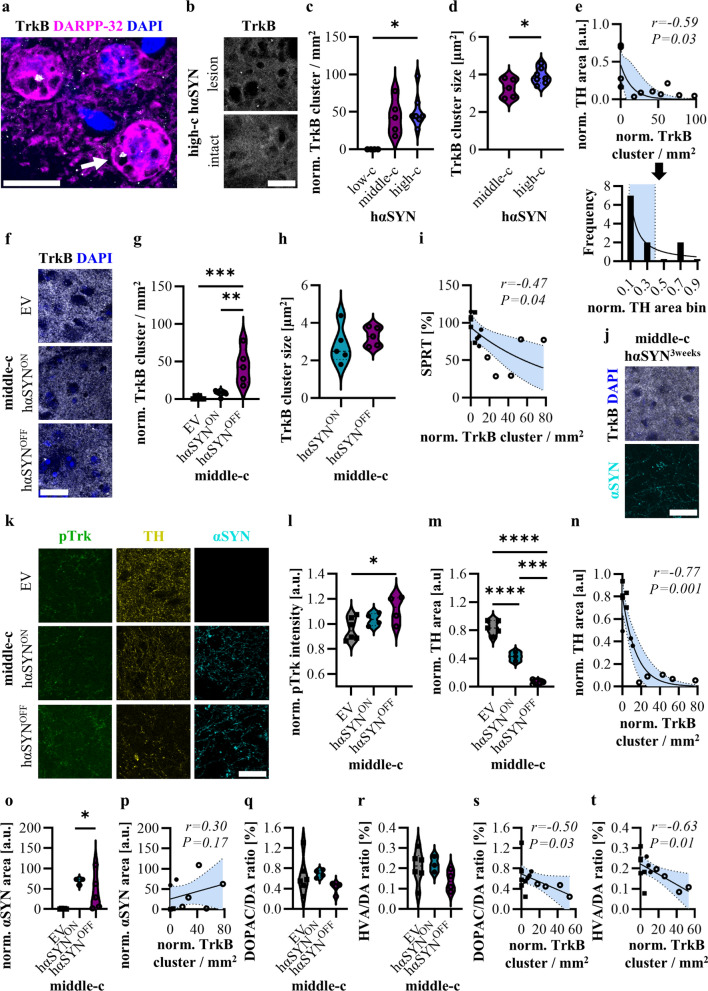
TrkB cluster formations in hαSYN rats and the effect of STN-DBS. **a** A confocal image of perinuclear TrkB cluster (arrow) in striatal MSNs. Scale bar: 20 μm. **b** Exemplary ROI of the lesioned and intact hemispheres of a high-c hαSYN animal. Scale bar: 50 μm. **c** Normalized TrkB cluster density comparison across hαSYN groups. Kruskal–Wallis test: H(2) = 8.909, *P* = 0.0044, *n* = 4–7. **d** Comparison of TrkB cluster size between middle-c and high-c hαSYN groups. Unpaired *t*-test:* t*(10) = 2.397, *P* = 0.0375, *n* = 5–7. **e** Pearson correlation of TrkB density and TH area. Histogram shows frequency distribution with 95% CI (0.056–0.392) (Black arrow). **f** Microscopy of perinuclear TrkB clusters in the lesioned striatum of middle-c animals. Scale bar: 50 μm. EV: empty vector. **g** TrkB density quantification. One-way ANOVA followed by Tukey’s multiple comparisons test: *F*_(2, 13)_ = 15.30, *P* < 0.001, *n* = 5–6. **h** TrkB cluster size in middle-c hαSYN^ON^ vs. hαSYN^OFF^. Unpaired *t*-test: *t*(8) = 0.9240, *P* = 0.3825, *n* = 5. **i** Pearson correlation of TrkB density with single pellet reaching task (SPRT) performance. **j** Microscopic images of TrkB, DAPI and αSYN signal in middle-c hαSYN^(3weeks)^ rats. Scale bar: 50 µm. **k** Microscopic images of pTrk, TH and αSYN in middle-c groups (separate stainings). Scale bar: 50 μm. **l** pTrk mean intensity in the lesioned hemisphere normalized to the intact hemisphere. One-way ANOVA: *F*_(2, 11)_ = 3.688, *P* = 0.0595, *n* = 4–6. **m** TH area within ROI in the lesioned hemisphere normalized to the intact hemisphere. One-way ANOVA: *F*_(2, 10)_ = 143.3, *P* < 0.0001, *n* = 3–5. **n** Pearson correlation of TrkB density and TH area. **o** αSYN area within ROI. One-way ANOVA: *F*_(2, 10)_ = 5.780, *P* = 0.0215, *n* = 3–5. **p** Pearson correlation of TrkB density and αSYN area. **q** DA turnover (DOPAC/DA ratio). One-way ANOVA: *F*_(2, 11)_ = 1.464, *P* = 0.2731, *n* = 4–6. **r** DA turnover (HVA/DA ratio). One-way ANOVA: *F*_(2, 10)_ = 1.762, *P* = 0.2210, *n* = 3–6. **s** Pearson correlation of TrkB density and DOPAC/DA ratio. **t** Pearson correlation of TrkB density and HVA/DA ratio. Squares, middle-c EV; Black dots, middle-c hαSYN^ON^; transparent dots, middle-c hαSYN^OFF^. HVA, Homovanilic acid; DOPAC, 3,4-Dihydroxyphenylacetic acid; DA, Dopamine

Considering the absence of TrkB clusters in low-c hαSYN rats and the potential toxicity of the high-c model, we next investigated the effect of STN-DBS on cluster formation in the middle-c hαSYN rats. Chronic stimulation was applied continuously over three weeks at 130-Hz frequency, 60-µs pulse length and individual amplitudes set 20% lower than the level exerting side effects. The TrkB cluster density of non-stimulated middle-c hαSYN^OFF^ rats was significantly higher compared to the empty-vector injected controls (middle-c EV). Intriguingly, STN-DBS reduced the density of TrkB clusters to a level comparable to that of the EV controls (Fig. 1f, g). No difference was observed in the cluster size between middle-c hαSYN^OFF^ and middle-c hαSYN^ON^ rats (Fig. 1h). Notably, the number of TrkB clusters correlated negatively with the motor performance in the single pellet reaching task (Fig. 1i). To evaluate if STN-DBS prevents TrkB cluster formation, TrkB quantification was performed in an additional group of middle-c hαSYN rats, three weeks post-AAV-injection, prior to chronic stimulation. No TrkB clusters were detected, despite αSYN accumulation already present in the striatum at this disease stage (Fig. 1j). We then measured levels of phosphorylated Trk (pTrk) indicating receptor activation in the same animal cohort. pTrk intensity was significantly higher in the hαSYN^OFF^ but not in the hαSYN^ON^ rats compared to EV controls (Fig. 1k, l). This observation suggests that STN-DBS prevents TrkB cluster accumulation and lowers pTrk levels in the striatum of PD rats.

In the hαSYN^OFF^ PD rats, dopaminergic fibers were significantly reduced in the striatum compared to EV controls, as indicated by the area covered by TH signal (Fig. 1k, m). Chronic STN-DBS over three weeks significantly restored dopaminergic fibers in the striatum of hαSYN PD rats (Fig. 1k, m). The amount of striatal dopaminergic projections (represented by the area of TH signals) negatively correlated with the TrkB cluster density (Fig. 1n). In contrast, the αSYN signal was similar between hαSYN^OFF^ and hαSYN^ON^ animals, indicating that STN-DBS did not affect the striatal αSYN level (Fig. 1o). In addition, αSYN profiles showed no correlation with TrkB cluster density (Fig. 1p), suggesting that the striatal αSYN level does not directly affect TrkB aggregate formation. Net dopamine turnover, reflecting dopaminergic neuron activity as well as striatal dopamine replacement and degradation rate, showed no significant differences between hαSYN^OFF^ with hαSYN^ON^ rats based on homovanilic acid/dopamine and 3,4-dihydroxyphenylacetic acid/dopamine ratios (Fig. 1q, r). However, TrkB cluster formation correlated negatively with dopamine turnover (Fig. 1s, t). These results suggest that STN-DBS rescues dopaminergic striatal projections and reduces TrkB cluster formation depending on functional dopamine levels.

In this study, we demonstrated the development of pathological TrkB clusters in a progressive PD model induced by AAV1/2-A53T hαSYN injection. These clusters are analogous to those observed in FACS (fluorescence-activated cell sorting) dMSN cultures, 6-hydroxydopamine rats, and human PD post-mortem tissues [1]. The AAV-A53T hαSYN model allows control over neurodegeneration and αSYN accumulation through viral vector concentration and post-injection duration. Our results revealed that certain degeneration threshold is required for TrkB cluster formation, which follows hαSYN aggregate appearance. Dopaminergic denervation of about 60% in the nigrostriatal pathway is required to induce TrkB clustering. These results align with a previous study showing that dopamine depletion impairs TrkB translocation to the cell surface in dMSNs, leading to TrkB cluster formation [1]. TrkB accumulation is also linked to reduced responsiveness to BDNF from cortico-striatal neurons.

STN-DBS showed a beneficial effect on striatal MSNs by reducing pathological TrkB aggregation in PD rats and preserving nigrostriatal dopaminergic projections. Dopaminergic input is essential for TrkB translocation to the cell surface of striatal MSNs [1], a process that determines MSN sensitivity to cortical BDNF. This mechanism mediates synaptic plasticity on cortico-striatal synapses, affecting structural complexity and motor learning [2]. Interestingly, pTrk was increased in PD rats. This may indicate that pTrk is part of these aggregates within multivesicular bodies, or intracellular Trk transactivation is upregulated, or dopamine depletion disrupts TrkB retraction from the plasma membrane of D2-expressing indirect pathway MSNs (iMSNs) [3], leading to an increase of phosphorylated TrkB at the cell surface.

Considering the association of mutated αSYN aggregation with lysosomal dysfunction [4] and reduced BDNF/TrkB expression, transport and signaling [5, 6], we analyzed αSYN as a potential mediator for TrkB cluster development. However, no correlation between striatal αSYN level and cluster formation was found. At three weeks post injections, we observed overexpression of αSYN, but no TrkB clusters. Furthermore, the protective effect of STN-DBS seems to be independent of striatal αSYN levels, consistent with a previous study demonstrating no changes in total αSYN and oligomer levels in the SN of hαSYN rats after STN-DBS [7]. αSYN is typically degraded via autophagy, whereas TrkB has a role in stimulating the retrograde pathway for autophagy in axons [8, 9]. The initial accumulation of αSYN followed by TrkB aggregates may indicate general impairment of the autophagic process. However, the specific influence of STN-DBS on TrkB clusters rather than on αSYN levels suggests that the degradation pathways for these proteins are regulated differently.

The unchanged functional dopamine levels in PD rats compared to EV controls (measured by dopamine turnover) despite a reduction in dopaminergic innervation of the striatum, indicates increased activity of the remaining dopaminergic neurons from the SN in these animals. In addition, abnormally high neuronal activity has been reported to result in excitotoxicity and subsequent cell death [10]. Here, early STN-DBS modulates neural activity within the nigrostriatal tract and the cortico-striatal circuit, and the altered activity preserves dopaminergic terminals from SN afferents. Thus, the changes in neural activity could play a role in the neuroprotective effects of STN-DBS, and the preservation of TrkB expression at the cell surface of dMSNs could maintain the capacity for plasticity at this synapse, which appears essential for motor learning [2, 11]. Interestingly, this protective effect was also seen when αSYN had already accumulated in the striatum. However, it is unclear whether these accumulations of αSYN represent aggregates or just elevated levels of the mutant protein. A limitation of this analysis is the αSYN antibody, which is not specific for aggregated forms of αSYN. However, normalization to the intact hemisphere eliminates the inclusion of physiological αSYN. Further investigations are required to determine the effect of TrkB cluster formations within the cortico-basal ganglia loop. This biomarker might help identify a time window in which STN-DBS is most beneficial for treating neuronal network-induced pathoplasticity in PD.

## Supplementary Information

**Additional file** **1**. **Supplementary Materials and Methods.**

## Data Availability

The experiment data that support the findings of this study are available upon reasonable request to the corresponding author.

## Abbreviations

BDNF: Brain-derived neurotrophic factor
CI: Confidence interval
DBS: Deep brain stimulation
dMSN: D1-expressing direct pathway medium spiny neuron
hαSYN: Human mutated A53T α-synuclein
iMSN: D2-expressing indirect pathway MSN
PD: Parkinson's disease
pTrk: Phosphorylated Trk
SN: Substantia nigra
STN: Subthalamic nucleus
TH: Tyrosine hydroxylase
TrkB: Tropomyosin receptor kinase B

## References

[1] Andreska T, Lüningschrör P, Wolf D, McFleder RL, Ayon-Olivas M, Rattka M, et al. DRD1 signaling modulates TrkB turnover and BDNF sensitivity in direct pathway striatal medium spiny neurons. Cell Rep. 2023;42(6): 112575. 37252844 10.1016/j.celrep.2023.112575

[2] Andreska T, Rauskolb S, Schukraft N, Luningschror P, Sasi M, Signoret-Genest J, et al. Induction of BDNF expression in layer II/III and layer V neurons of the motor cortex is essential for motor learning. J Neurosci. 2020;40(33):6289–308. 32651187 10.1523/JNEUROSCI.0288-20.2020 PMC7424868

[3] Ayon-Olivas M, Wolf D, Andreska T, Granado N, Lüningschrör P, Ip CW, et al. Dopaminergic input regulates the sensitivity of indirect pathway striatal spiny neurons to brain-derived neurotrophic factor. Biology. 2023;12(10):1360. 37887070 10.3390/biology12101360 PMC10604681

[4] Mazzulli JR, Zunke F, Isacson O, Studer L, Krainc D. alpha-Synuclein-induced lysosomal dysfunction occurs through disruptions in protein trafficking in human midbrain synucleinopathy models. Proc Natl Acad Sci U S A. 2016;113(7):1931–6. 26839413 10.1073/pnas.1520335113 PMC4763774

[5] Kohno R, Sawada H, Kawamoto Y, Uemura K, Shibasaki H, Shimohama S. BDNF is induced by wild-type alpha-synuclein but not by the two mutants, A30P or A53T, in glioma cell line. Biochem Biophys Res Commun. 2004;318(1):113–8. 15110760 10.1016/j.bbrc.2004.04.012

[6] Volpicelli-Daley LA, Gamble KL, Schultheiss CE, Riddle DM, West AB, Lee VM. Formation of alpha-synuclein Lewy neurite-like aggregates in axons impedes the transport of distinct endosomes. Mol Biol Cell. 2014;25(25):4010–23. 25298402 10.1091/mbc.E14-02-0741 PMC4263445

[7] Lee EJ, Aguirre-Padilla DH, Fomenko A, Pawar G, Kapadia M, George J, et al. Reduction of alpha-synuclein oligomers in preclinical models of Parkinson’s disease by electrical stimulation in vitro and deep brain stimulation in vivo. Brain Stimul. 2024;17(2):166–75. 38342364 10.1016/j.brs.2024.02.005

[8] Webb JL, Ravikumar B, Atkins J, Skepper JN, Rubinsztein DC. Alpha-synuclein is degraded by both autophagy and the proteasome. J Biol Chem. 2003;278(27):25009–13. 12719433 10.1074/jbc.M300227200

[9] Sidibe DK, Kulkarni VV, Dong A, Herr JB, Vogel MC, Stempel MH, et al. Brain-derived neurotrophic factor stimulates the retrograde pathway for axonal autophagy. J Biol Chem. 2022;298(12): 102673. 36336077 10.1016/j.jbc.2022.102673 PMC9768381

[10] Verma M, Lizama BN, Chu CT. Excitotoxicity, calcium and mitochondria: a triad in synaptic neurodegeneration. Transl Neurodegener. 2022;11(1):3. 35078537 10.1186/s40035-021-00278-7 PMC8788129

[11] Park H, Poo MM. Neurotrophin regulation of neural circuit development and function. Nat Rev Neurosci. 2013;14(1):7–23. 23254191 10.1038/nrn3379

