# Supplementary material for: Chronic subthalamic nucleus deep brain stimulation reduces pathological TrkB aggregates in a Parkinson’s disease rat model
Source: Transl Neurodegener. 2025 Feb 20;14:11. doi: 10.1186/s40035-025-00472-x (PMC11843761; doi:10.1186/s40035-025-00472-x)
Supplement: Supplementary file 1 — Additional file 1. Supplementary Materials and Methods. [file 40035_2025_472_MOESM1_ESM.pdf]

Chronic subthalamic nucleus deep brain stimulation reduces pathological TrkB aggregates in a Parkinson's disease rat model

Tobias Petschner[1], Katarina Hofman[1], Jia Zhi Chen[1], Thomas Andreska[2], Daniel Wolf[2], Susanne Knorr[1], Robert Blum[1], Muthuraman Muthuraman[1], Uwe Gbureck[3], Jens Volkmann[1], Michael Sendtner[2] and Chi Wang Ip[1§]

[1]Department of Neurology, University Hospital of Würzburg, Josef-Schneider-Straße 11, 97080 Würzburg, Germany

[2]Institute of Clinical Neurobiology, University Hospital of Würzburg, Versbacherstraße 5, 97078 Würzburg, Germany

[3]Department for Functional Materials in Medicine and Dentistry, University Hospital Würzburg, Pleicherwall 2, 97070 Würzburg, Germany

[§]Correspondence to: Chi Wang Ip, Ip_C@ukw.de

Supplementary Material and Methods

Animal model

Adult male Sprague-Dawley rats were purchased from Charles River Laboratories (Sulzfeld, Germany). The animals were maintained under standard conditions (21°C, 12 h light/dark

cycle). We did not use female rats, because the described methodological aspects were independent of gender. All animal experiments were approved by the local government and performed in accordance with all applicable international guidelines for care and use of laboratory animals (Directive 2010/63/EU for animal experiments).

Animals received unilateral microinjection of either AAV1/2-expressing human mutated A53T-αSYN or empty AAV1/2 vector (EV) into the SN, similar to previous studies[1,2]. In short, anaesthetized rats were injected with 2 µl viral vector at the coordinates anterior-posterior (AP) -5.2 mm, medio-lateral (ML) +/-2.0 mm and dorso-ventral (DV) -7.4 mm related to bregma. Representative for different severity levels of hemiparkinsonian rats, one of three hαSYN vector concentrations was injected: 2.55×10^{12} genomic particles (gp)/ml (= low-c), 5.1×10^{12} gp/ml (= middle-c) or 15.3×10^{12} gp/ml (= high-c). Animals were perfused 6-8 weeks post-injection. Three weeks post-injection, the middle-c animals underwent an ipsilateral electrode implantation, but only DBS-ON animals received chronic stimulation for three weeks, similar to previous studies[1]. In short, the platinum/iridium electrode was implanted into anaesthetized rats with the tip targeting the STN at the coordinates AP -3.6 mm, ML +/-2.5 mm and DV -7.7 mm related to bregma. An additional group of middle-c animals was perfused 3 weeks post-injection to characterize the state at the time point before similarly treated animals received stimulation. The multiplicity of conditions resulted in the following groups: low-c hαSYN; middle-c EV; middle-c hαSYN (hαSYN^{OFF}); middle-c hαSYN^{ON}; middle-c hαSYN$^{(3weeks)}$; high-c hαSYN.

Tissue Processing and Immunohistochemistry

After transcardial perfusion with 0.34% heparin sodium-25000-ratiopharm (Ratiopharm #10108193648610) in 0.1 M phosphate-buffered saline (PBS), the brains were incubated in 4% paraformaldehyde (PFA; Merck #818715) in 0.1 M PBS for 48 hours for an adequate

immersion fixation. The tissue processed for immunohistochemistry (IHC) was then transferred into 30% sucrose (Roth #4621.1) and 0.1 M PBS solution (72 h) before freezing and coronal sectioning in 40 µm slices. Three striatal slices at the same positions between +1 and -2.5 mm related to bregma were selected from each animal for the analysis. In certain cases, the number of selected slices was reduced, due to low tissue quality.

Brain slices were washed in 0.1 M PBS, followed by 2 h incubation time in a blocking solution consisting of 0.1 M PBS, 10% normal donkey serum (NDS; Merck #S-30), 0.3% Triton X-100 (Sigma #X100), 0.1% Tween20 (Sigma #P9416) and 0.1% NaN_3 at room temperature (RT). Afterwards, first antibodies were added to the blocking solution. The following first antibodies were applied for three days at 4°C: Rabbit anti-αSYN (Sigma #S3062; RRID: AB_477506; c = 1 µg/ml); chicken anti-TH (Abcam #ab76442; RRID: AB_1524535; c = 0.2 µg/ml); goat anti-TrkB (R&D Systems #AF1494; RRID: AB_2155264; c = 0.44 µg/ml); rabbit anti-DARPP32 (Cell Signaling Technology #2306; RRID: AB_823479; c = 0.43 µg/ml). Dopamine and cyclic AMP regulated phosphoprotein of 32 kDa (DARPP32) and αSYN were labeled separately. Slices were washed again in 0.1 M PBS before incubating in blocking solution containing secondary antibodies for 2 h at RT: Donkey anti-goat Cy3 (Jackson Immunoresearch #705-165-147; RRID: AB_2307351; c = 1.25 µg/ml); donkey anti-rabbit AF488 (Jackson Immunoresearch #711-545-152; RRID: AB_2313584; c = 1.25 µg/ml); donkey anti-chicken AF647 (Jackson Immunoresearch #703-605-155; RRID: AB_2340379; c = 0.625 µg/ml). After another washing step, 4′,6-diamidino-2-phenylindole (DAPI) in 0.1 M PBS was applied for 15 min at RT. Finally, the brain slices were washed and embedded in Aqua-Poly/Mount.

For a separate labeling of phosphorylated Trk (pTrk), we performed an antigen retrieval with 10 mM sodium citrate (Sigma #S4641) and a heating step at 80°C for 30 min. Afterwards, slices were processed as described above. We used rabbit anti-pTrk PLC-gamma (Sigma

#ABN1381; RRID: AB_2721199; c = 1 µg/ml) as the primary antibody and donkey anti-rabbit Cy5 (Jackson Immunoresearch #711-175-152; RRID: AB_2340607; c = 0.55 µg/ml) as the secondary antibody.

The following combinations of proteins were labeled in separate stainings: TrkB + αSYN or TH + DAPI; TrkB + DARPP32 + DAPI; pTrk.

Confocal microscopy and image analysis

Confocal images were acquired using an Olympus IX81 microscope, equipped with an Olympus FV1000 confocal laser-scanning system and a FVD10 SPD detector. Diode lasers (405, 473 and 559 and 635 nm) were used for fluorophore excitation. The software Olympus FV10-ASW (RRID: SCR_014215) enabled scanning modes and image acquisition.

First, regions of interest (ROI) of each coronal brain slice were defined. Here, five striatal ROI of each hemisphere were selected, focusing on dorsal areas near the corpus callosum, which receive dense afferent input from the motor cortex[3-5], as described previously[6]. Using an Olympus UPLSAPO 60x (oil; numerical aperture: 1.35) objective, we imaged ROI with a length of 212 µm, a width of 212 µm, a depth of 3.75 µm (5 x 0.75 µm step interval) and a resolution of 3.77 px/µm. To capture the different fluorescence-coupled marker, the pre-installed channels for Cy3, AF488, AF647 and DAPI were employed. The software Olympus FV10-ASW (RRID: SCR_014215) enabled scanning modes and image acquisition.

Next, the software ImageJ (RRID: SCR_003070) was used for image processing and analysis. For the TrkB cluster identification, the display range of the Cy3 channel images was set to 12-bit (grey level range of 0–4095). Each layer of the Z-stack was explored to detect TrkB cluster formations. Afterwards, the Cy3 channel was merged with the DAPI channel to verify the perinuclear localization of TrkB clusters. The number of clusters was calculated per mm^2 to

present the TrkB cluster density. The image depth was only used for better cluster identification and visualization, but disregarded in this calculation. The average cluster density of the lesioned hemisphere, ipsilateral to the viral vector injection, was normalized to the intact hemisphere. Additionally, the two-dimensional area of clusters was determined using the polygon tool and area measurement function after a maximal intensity Z-stack projection. For these analyses, two striatal brain slices with 10 ROI each were investigated.

Tyrosine hydroxylase (TH) and αSYN was quantified by determining the signal covered area after background subtraction and the auto-threshold Otsu segmentation. The average signal covered area within the ROI is a readout for the amount of TH^+ fibers or αSYN. The pTrk signal was quantified by the mean fluorescence intensity after background subtraction, which describes the mean grey value of each ROI. An intensity-related analysis was selected instead of an area-related analysis, because pTrk signal was expected to be naturally widely distributed across striatal MSNs and more likely varied in intensity. A prior background subtraction was required. Few animals were excluded from this analysis due to low tissue quality.

Measurement of dopamine turnover by HPLC

Analysis of homovanilic acid (HVA), 3,4-Dihydroxyphenylacetic acid (DOPAC) and DA was conducted using high-performance liquid chromatography (HPLC) as previously described[1]. The investigator was blinded to the experimental groups and treatment conditions to ensure unbiased results. Brain sections were homogenized and then subjected to centrifugation at 10,000 g for 20 minutes. HVA, DOPAC and DA were subsequently quantified from the supernatant. The concentrations are reported as nanograms of analyte per milligram of total protein.

Single pellet reaching task

The reaching assessment was performed and analyzed as described earlier[1]. First, the dominant forepaw of each rat was assessed. Viral vector injection and electrode implantation were then performed contralaterally to the dominant forepaw. The motor performance of rats eight weeks post-injection is presented as a percentage of the individual preoperative performance. All animals were scored under stim-OFF conditions.

Statistical Analysis

Statistical analysis was performed using Graph Pad Prism version 9.1.2. Normality was tested by Q-Q-plots. Normal distributed data sets comparing multiple groups were statistically analyzed by one-way ANOVA and Tukey's multiple comparisons test. Non-parametric data sets comparing multiple groups were analyzed using the Kruskal–Wallis test followed by Dunn's multiple comparisons test. Normal distributed data of two groups was statistically analyzed by unpaired t-test. Correlation was determined by Pearson correlation tests. (*) $P < 0.05$, (**) $P < 0.01$, (***) $P < 0.001$, and (****) $P < 0.0001$ were considered significant p values. A regression curve with a 95% confidence interval was generated and visualized. Data normality was assessed, and distribution analyzed using an XY-plot. Model suitability was evaluated by the coefficient of determination (R^2) and compared to alternative models.
